# Ionotropic Gelation and Chemical Crosslinking as Methods for Fabrication of Modified-Release Gellan Gum-Based Drug Delivery Systems

**DOI:** 10.3390/pharmaceutics15010108

**Published:** 2022-12-28

**Authors:** Piotr Gadziński, Anna Froelich, Barbara Jadach, Monika Wojtyłko, Adam Tatarek, Antoni Białek, Julia Krysztofiak, Michał Gackowski, Filip Otto, Tomasz Osmałek

**Affiliations:** 1 Chair and Department of Pharmaceutical Technology, Poznan University of Medical Sciences, 6 Grunwaldzka Street, 60-780 Poznań, Poland; 2 Doctoral School, Poznan University of Medical Sciences, 70 Bukowska Street, 60-812 Poznań, Poland; 3 Student’s Research Group of Pharmaceutical Technology, Chair and Department of Pharmaceutical Technology, Poznan University of Medical Sciences, 6 Grunwaldzka Street, 60-780 Poznań, Poland

**Keywords:** gellan gum, ionotropic gelation, modified release, bioavailability, drug delivery

## Abstract

Hydrogels have a tridimensional structure. They have the ability to absorb a significant amount of water or other natural or simulated fluids that cause their swelling albeit without losing their structure. Their properties can be exploited for encapsulation and modified targeted drug release. Among the numerous natural polymers suitable for obtaining hydrogels, gellan gum is one gaining much interest. It is a gelling agent with many unique features, and furthermore, it is non-toxic, biocompatible, and biodegradable. Its ability to react with oppositely charged molecules results in the forming of structured physical materials (films, beads, hydrogels, nanoparticles). The properties of obtained hydrogels can be modified by chemical crosslinking, which improves the three-dimensional structure of the gellan hydrogel. In the current review, an overview of gellan gum hydrogels and their properties will be presented as well as the mechanisms of ionotropic gelation or chemical crosslinking. Methods of producing gellan hydrogels and their possible applications related to improved release, bioavailability, and therapeutic activity were described.

## 1. Introduction

Delivery of the active pharmaceutical ingredient (API) to the specific site of the gastrointestinal tract (GIT) and its release in a controlled and predictable manner have been one of the most important challenges for pharmaceutical technology for the past few decades. Many drugs applied orally are either sensitive to the acidic environment of the stomach or cause moderate to severe side effects in the proximal regions of GIT [1,2,3]. Such disadvantages may contribute to lower therapeutic efficiency or lead to reluctant use by the patients. To avoid this, the drug release often needs to be modified and shifted to the distal parts of the GIT, which is usually achieved by coating with pH-sensitive polymers or incorporation into polymeric matrices [4,5]. In the case of some intestinal malfunctions, it is necessary not only to deliver the drug to the site of action but also to extend the release as much as possible to provide proper API concentration in time. As the targeted drug delivery lies within the field of pharmaceutical technology, various formulations have been prepared and investigated to face this challenge. These include liposomes, micro-, nanoparticles, emulsions, or suspensions [6,7,8,9]. As the targeted delivery to the GIT is mostly considered to treat various inflammatory bowel diseases (IBD), Crohn’s disease, ulcerative colitis, or colorectal cancer, therapeutic strategies aim to prevent the recurrence of inflammation and maintain remission. Therefore, such formulations usually contain corticosteroids, which are considered the most effective treatment of the active phase of IBD, mesalazine, azathioprine, 6-mercaptopurine, or antibiotics (e.g., ciprofloxacin, metronidazole). However, a proper oral dosage form should be characterized by easy handling to the patient, accurate dose, good stability, safety, and low production costs. Zhang et al. evaluated the potential of diatom silica obtained from single-cell photosynthetic algae for the delivery of substances that are poorly soluble in water, such as mesalamine and prednisone, which are commonly prescribed for ulcerative colitis treatment. They have also evaluated cytotoxicity on Caco-2, HT-29, and HCT-116 cells. Results showed that diatoms have exceptionally low toxicity even in high concentrations (up to 1000 µg/mL). What is more, mesalamine and prednisone indicated prolonged release from diatoms in simulated GI conditions. Furthermore, permeation of the API across the Caco-2/HT-29 co-culture monolayers showed that diatoms can enhance the permeability of poorly soluble drugs. The outcome of this study is that diatoms can be considered proper, nontoxic mesalamine and prednisone carriers with the ability to improve their bioavailability and provide prolonged release [10]. Tang et al. made an attempt to develop a new modified-release dosage form for mesalamine based on nanoparticles containing hydroxypropyl-β-cyclodextrin (HP-β-CD) inclusion complex loaded chitosan (CS). Compared to free 5-aminosalicylic acid (5-ASA), these nanoparticles showed a sustained release behavior. Anti-inflammatory activity was defined with the use of HT-29 cell lines stimulated with cytokines. The observed parameters were inflammatory mediators. The results showed stronger inhibition of the production of inflammatory mediators in the case of the prepared nanoparticles. Therefore, HP-β CD/5-ASA/CS nanoparticles (NPs) possibly revealed a higher potential to reduce inflammation and may be a good delivery system of mesalamine with a sustained release effect [11].

Unfortunately, the majority of conventional non-targeted dosage forms available on a market can exhibit unwanted side effects, and their efficacy can be decreased because of the absorption of the drug to the bloodstream before reaching the target site. Furthermore, novel dosage forms are often either hard to transfer from the manufacturing process to the industrial scale or very expensive. Processing innovative dosage forms with improved characteristics in comparison to commonly used formulations is highly challenging and a very current issue. There is also no doubt that the discovery of new materials, or modification of those already used, often sets completely new directions in the development of innovative pharmaceutical formulations. However, it should be clearly stated that the introduction of new pharmaceutical excipients is associated with the need for a very detailed description and assessment of their properties, aimed at providing effective, but above all safe therapy. 

Therefore, natural-polymer-based hydrogels obtained with ionotropic gelation or/and chemical crosslinking seem to be the current trend of searching for new carriers for known active substances to improve their effectiveness and increase the safety of the therapy. According to widespread availability, lack of or very low toxicity, biodegradable properties, and relatively low production costs, naturally derived polymers have highly influenced the food industry, personal care products, and pharmaceutical technology [12,13,14]. Recent studies showed the immense potential of dosage forms based on natural polymers. Generally, high drug encapsulation rate, pH-dependence behavior, and simple manufacturing process make them suitable candidates to form a proper drug carrier. 

Among natural polymers used for this purpose, gellan gum (GG) is one with currently increasing interest in potential applications in pharmaceutical technology. Its ability to undergo a sol–gel transition and to form a solid gel upon contact with cations, which is named ionotropic gelation, may be used to prepare drug-loaded matrices [15]. It was discovered in the lab of Kelco company, a division of Merck, in 1978 [16]. It was first isolated from non-pathogenic aerial Gram-negative bacteria, *Sphingomonas elodea*, formerly known as *Pseudomonas elodea* [17]. The first use of gellan gum was mentioned in the case of replacement of the agar in microbiology laboratories. Gellan gum is a linear polysaccharide characterized by a high molecular weight of approximately 1.0 kg/mol and is built by a repeated tetrasaccharide composed of α-L-rhamnose, β-D-glucuronic acid, and β-D-glucose, in the molar ratios of 1:2:1 [18]. It comes in two types: native (high-acyl gellan, HA gellan) and deacetylated (low-acyl gellan, LA gellan). HA gellan contains two types of constituents attached to β-D-glucose. It is an L-glycerol moiety at the C2 atom and an acetyl group at atom C6. LA gellan occurs as a result of the effect of alkaline hydrolysis. The LA gellan is broadly investigated as a component of oral drug delivery systems with the sustained or modified release [19,20,21,22]. In practice, the encapsulation of drugs into gellan gum matrices can be performed in two ways. In the first method, the gelling ions (most often Ca^2+^) solution is instilled drop-wise into the mixture of a drug and a polymer. Then, the spherical capsules with the liquid interior are obtained and the drug is located in the shell. The second way is to instill the drug–polymer mixture into the solution of ions, which results in the formation of matrix capsules (beads). The drug is then located in the whole sphere. After dehydration, the beads or capsules can mostly be stored in a dry environment or administered to the patient. In the case of drug encapsulation into gellan matrices, the properties of the final product (shape, uniformity, hardness) are strongly related to the conditions during production. Moreover, drug release is the result of multiple processes among which polymer and crosslinker concentrations, swelling, and polymer network degradation are regarded as the most important. In turn, they depend on the gelation course, but also on the composition of the matrices and the addition of other components [18,23]. Recent studies have shown the great potential of dosage forms based on natural polymers. 

However, some papers indicate that gellan matrices stabilized with cations only may be characterized by insufficient mechanical resistance, which may result in too rapid degradation in the digestive tract and the release of the encapsulated drug before the target site [24,25]. Given the above, considerable efforts have been directed toward various methods and techniques for improving the three-dimensional structure of the gellan hydrogel. Chemical crosslinking seems to be an appropriate and very promising tool to achieve this goal [26]. Modification of gellan matrices using glutaraldehyde (GA) significantly increases their stability in a neutral or slightly alkaline environment [27]. 

In this paper, we would like to present a comprehensive review of methods for obtaining gellan gum-based drug delivery systems: ionotropic gelation and chemical crosslinking. Processes, mechanisms, devices, and gelling agents were described thoroughly. Above all, oral dosage forms for a modified and targeted release were described, but in situ gelling dosage forms for topical use were also mentioned. 

## 2. Ionotropic Gelation

Ionotropic gelation is a phenomenon exploiting the ability of polyelectrolytes (e.g., polysaccharides) to react with oppositely charged molecules (e.g., cations) and undergo the sol–gel transition, which results in the forming of structured physical materials (films, beads, hydrogels, nanoparticles). Recently, there has been a constant increase in interest in polysaccharide-based hydrogels for API encapsulation. A couple of techniques can be applied for hydrogel formation. Many of them are based on the preparation of spherical or oval beads composed of API and polymeric excipients. Crucial parameters determining the size and shape of droplets are the viscosity of the initial mixture, the surface tension, as well as the dynamic interactions between droplets and the matrix fluid (laminar or turbulent flow) as well as polymer concentration and molecular weight of the polymer.

### Mechanisms of Ionotropic Gelation 

In general, ionotropic gelation occurs between oppositely charged molecules. Positively charged polymer chains react with negatively charged divalent or multivalent ions. The electrostatic reaction leads to forming of the microstructured particles with interconnected nano-fibrillar networks. Such a structure can be achieved using three separate methods: internal, external, or inverse gelation [28]. 

The most frequently used method of ionotropic gelation is the external method. It is also known as a controlled diffusion method. The solution of a polysaccharide is instilled dropwise to the crosslinking solution. The bead matrix is obtained by the diffusion of crosslinking agents from the outer continuous phase into the inner structure of the polymer droplets. In the external layer of the formed hydrogel bead, the sol–gel transition process is fast, and gel formation is immediate. In the next steps, the counter-ions begin to penetrate through the next layers into the middle of the particle, creating a heterogeneous gelation profile in which the interaction between the ions and the polymer functional groups is maximum at the surface and zero at the core [29,30,31]. 

Internal gelling is also called in situ gelling. This approach is also widely described as a method for the preparation of polymer particles. In this case, the insoluble calcium salt (e.g., CaCO_3_ and CaSO_4_) is mixed with the polymer solution, and the obtained mixture is then extruded into an acid crosslinking bath. The altered conditions increase the solubility of the calcium salt, allowing it to be released, leading to the formation of a gel network of the polysaccharide. This allows the gelling mechanism to be controlled and the polymer to be uniformly exposed to cations, thereby creating a uniform gel network. The downside of this method is that the obtained matrices are characterized by lower density and larger pore sizes; therefore, they are more permeable than those obtained by external gelling, which results in lower entrapment efficiency and higher release rates. This is due to the increased permeability of the matrix, which is influenced by competition between Ca^2+^ and H^+^ ions due to the added acid. It appears that although the acid in the gelling bath improves the solubility of calcium salts, it also competes with Ca^2+^ for interaction with the polymer. This problem can be solved by changing the pH of the gelling medium and the amount of calcium ions donors used [32,33].

**Figure 1:**
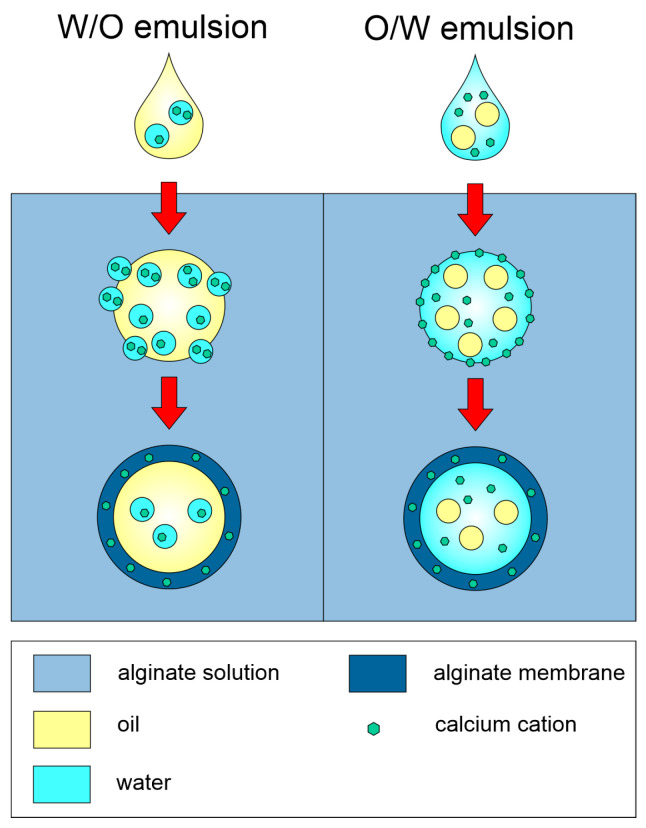
Reverse gelation scheme depending on emulsion type used.

Another approach is reverse gelation, which is based on the dropwise addition of a medium containing gelling agents to the polymer solution. Such a method can be typically used in the case of emulsions for the preparation of polymer-based soft-shell microcapsules with oil content. This method uses small amounts of biopolymer, resulting in forming of a soft molecular shell. The distinctive features of the microcapsules obtained (e.g., mechanical properties and release of API) may vary depending on the type of emulsion used (W/O or O/W), as shown in Figure 1 [34,35,36]. 

## 3. Other Methods of Gelation

### 3.1. Covalent (Chemical)

In the chemical crosslinking method, hydrogels are obtained by covalent crosslinking, which creates strong chemical networks. The biggest drawback is that most of the chemical cross-linkers do not ensure biocompatibility [37]. Among them, glutaraldehyde, for sure, is most often described; it is broadly used as a crosslinking agent for several biopolymers. Among them, chitosan [38] sodium alginate [39], cellulose [40], or gellan gum [41] can be distinguished. For example, polymer microspheres can be prepared by mixing solutions of polymer and glutaraldehyde in a mixture of oil and surfactants. This results in a Schiff base reaction occurrence between an aldehyde and an amine group. As the outcome of this reaction, polymer chains are covalently bonded with glutaraldehyde molecules.

### 3.2. pH-Induced

pH-Induced gelation can be triggered by varying the pH value of polymer solutions. Each drop of a polymer solution starts to gel upon contact with an acidic or alkaline crosslinking medium. In the first step, the shell is formulated. Next, ions diffuse through the shell, and the process of gelation continues. This method can be used alone or combined with other gelling methods as well to obtain the hydrogel particles [42,43].

### 3.3. Temperature-Induced

Temperature-induced sol–gel transition is also known as thermotropic or cryo-gelation. This method uses the ability of polymer chains to undergo the association into the more oriented form, e.g., coil to helix, and then into a double helix in response to temperature. The process takes place mostly after decreasing the temperature. The combination of these helices leads to the formation of a double helix and then to a gel network [44,45]. 

### 3.4. Non-Solvent

**Figure 2:**
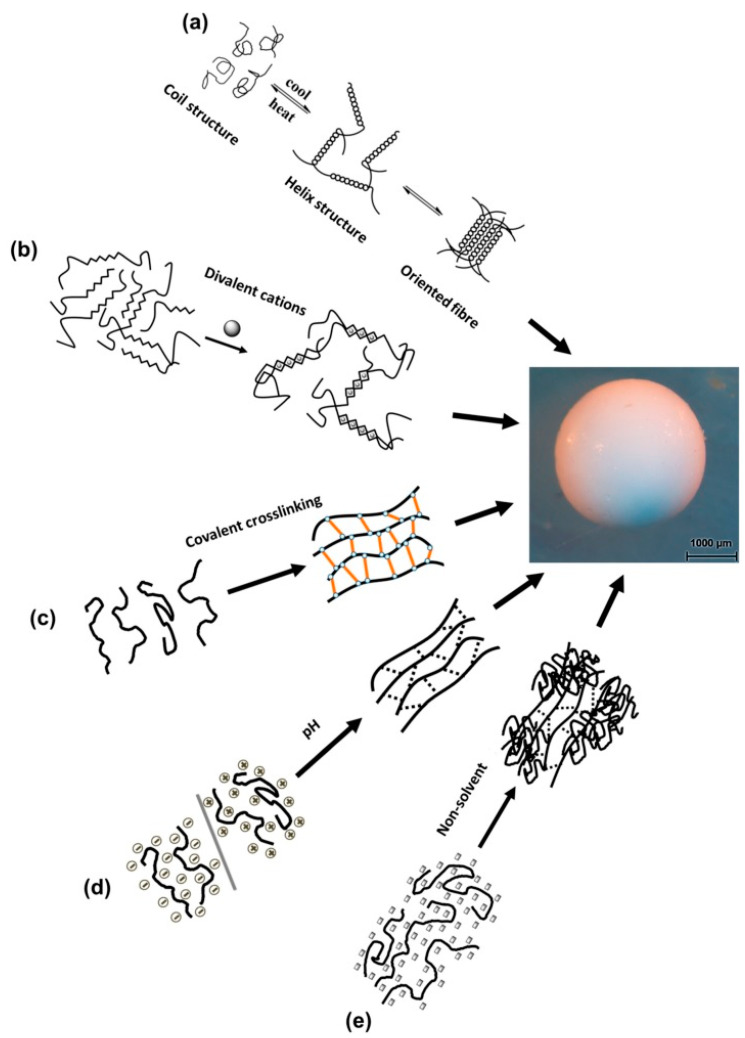
Schematic illustration of the mentioned mechanisms of formation of polymer hydrogel particles: (**a**) temperature-induced (thermotropic) gelation in which the polysaccharides undergo the structural transition from the coil to helix and then to double helix, (**b**) ion-induced (ionotropic) gelation in which the polysaccharide molecules are crosslinked by ions, (**c**) covalent crosslinking approach in which the polysaccharide chains are covalently crosslinked to form gel network, (**d**) pH-induced gelation, and (**e**) non-solvent approach to producing a non-solvent filled gel network [37].

Non-solvent induced phase separation (NIPS) is also called a process of coagulation or immersion precipitation. This method uses the solution of a polymer in a certain solvent which is pressed into a gelling bath containing a non-solvent. After that, a sharp decrease in the solubility of the polymer takes place, causing phase separation. The polymer chains combine to form a self-contained 3D network with the non-solvent. In general, the addition of non-solvent leads to the shrinking of the polysaccharide macromolecules. Its structure does not completely collapse if the polymer concentration is above the loading concentration [46,47]. Schematic illustrations of mentioned methods of gelation are presented in Figure 2.

## 4. Formation of Droplets

### 4.1. Conventional Dropping Method 

Droplet-producing devices are used mainly on a laboratory scale. This method consists of manually extruding polymer droplets by using a syringe or pipette filled with polymer and API solution into a gelling agent mixture. These drip devices produce droplets at the sharp end (orifice) of the needle. Tear-shape drops free-falling into the gelling agent solution is caused by the influence of gravity [28]. The polymer droplet enlarges until it detaches from the hole by gravity and falls towards the gelling medium. Since besides gravity, there are no further driving forces to cause a drop to fall from the orifice, these devices produce large droplets of a few millimeters in size, which are usually larger than the diameter of the nozzle. Important parameters determining the size of the droplets are the viscosity of the liquid, polymer concentration, polymer molecular weight, and the diameter of the nozzle. In conventional drip methods, a couple of physicochemical factors such as surface tension, density, dynamic viscosity and process parameters such as nozzle geometry or gravity can influence the size and shape of obtained particles as well. With these dumping devices comes a serious drawback, which is exceptionally low production capacity. A potential solution to this problem may be multiplying the number of nozzles, which can significantly improve the lab-scale droplet production capacity. Some described devices use coaxial gas flow around the nozzle. This is not the atomization process, but such action can improve the rate of particle detachment from the orifice, resulting in a decrease in particle size compared to the conventional drop method. Flowing gas supports the force of gravity gently shearing the nozzle tip and pushing the mixture out of the orifice. This method should be applied when droplets revealing a diameter smaller than 1 mm are desired [48,49,50].

### 4.2. Vibrating Nozzle-Prilling

This method uses a laminar stream of an initial mixture flowing through the nozzle, to which frequency-optimized overlapping vibrations are applied to form monodisperse droplets. These vibrations can be generated utilizing sound waves [51,52]. The initial mixture composed of polymer and API is exposed to a pump or gas through a nozzle to create a liquid jet. Superimposed vibrations destabilize the liquid jet (Rayleigh instability), and then the jet is crushed into monodisperse tear-shape droplets. In the literature, such a technique is also named the granulation method [52,53,54,55]. A couple of factors can influence the shape, size, and size distribution of the droplets and, consequently, the resulting hydrogel particles. Among them, the density, dynamic viscosity, and flow rate of the feed solution, the geometry, the diameter of the nozzle, the vibration frequency, and the falling distance can be named. Viscosity is, for sure, one of the most important factors that need to be considered in this technique; prilling can be applied only for solutions characterized by a viscosity below several hundred mPa⋅s [55]. Moreover, the sphericity of the particles can be significantly varied by changing the distance between the nozzle and the crosslinking bath. Upon droplets’ contact with the surface of the gelling medium, their spherical shape may become deformed. If the droplet’s viscosity and its surface tension are significantly lower than the surface tension of the gelling agent, it can affect the spherical shape of the droplet. Various studies have shown that liquid droplets of polymer solution are, in general, able to overcome the impacts that form spherical gel particles when the falling distance is greater than 10 cm [49].

### 4.3. Electrostatic

In the electrostatic method droplets, forming can be amplified by using an electric field as the initial mixture is forced through the charged matrix. Such a field draws the mixture in the form of droplets from the outlet of the orifice. An induced electrostatic charge is applied to the surface of the polymer solution droplets when it is torn out of the orifice. Coalescence is prevented via the electrostatic repulsion of liquid droplets in the gaseous phase. Droplet disintegration and droplet size are dependent on parameters such as mixture viscosity, nozzle diameter, distance to the gelling bath, and applied voltage. The obtained droplet size can be decreased to tens of micrometers by reducing the nozzle diameter, decreasing the distance between the electrodes, and increasing the applied voltage [56,57]. Processing efficiency can be improved by applying a multi-nozzle system. Such droplet formation can be compared to electrostatic atomization. However, unlike electrostatic spraying, in which overpressure is used, liquid droplets are formed from a single, low-velocity jet at the exit of a narrow nozzle. This method is characterized by producing droplets of very similar size. The applied electric potential can be static or pulsating. Among the drawbacks of this method, a limitation to low-viscosity liquids has to be mentioned [57].

### 4.4. Mechanical Cutting

**Figure 3:**
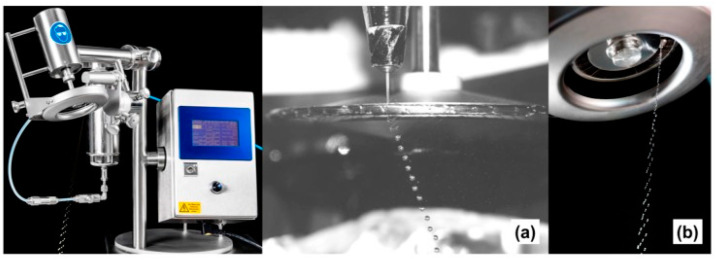
Image showing the tabletop JetCutter machine on the left and different Jet cutting tools producing a single stream of droplets: single (**a**) and multi-stream of droplets (**b**) [37].

**Figure 4:**
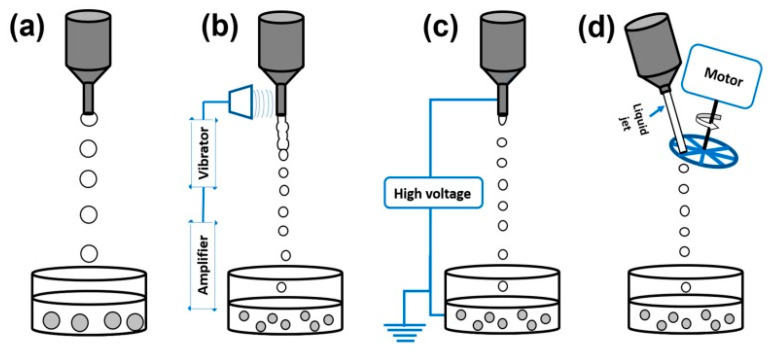
Illustration of dropping devices: (**a**) conventional dropping method influenced by gravity, surface tension, and viscosity; breaking up of liquid jets into droplets stimulated by (**b**) vibrating nozzle method, (**c**) electrostatic forces, and (**d**) a mechanical cutting device [37].

The production of particles ranging in size from several hundred micrometers to several millimeters with high efficiency and economic efficiency is possible due to mechanical cutting technology. The point of this technology is based on the mechanical cutting of a continuous stream of the initial mixture. The polymer solution is pumped through the nozzle at high speed to obtain a steady stream of liquid. In the next step, a rotating tool with strings/wires cuts the stream of liquid into cylinders of equal size, which, as they fly through the air, become almost spherical droplets as they fall into the gelling bath. This technique enables the preparation of spherical particles of specific size by varying parameters such as nozzle diameter, cutting frequency, and jet velocity. Comparing it to the classic dripping method, it has to be noted that mechanical cutting is characterized by high production speed and gives a possibility to work with highly viscous liquids, dispersions, and alloys, preferably thinned by shear (viscosity from 0.2 Pa⋅s to 110 Pa⋅s) [58]. The cutting technique is very much preferred for producing particles ranging from 0.2 to 0.8 mm. Two factors affect productivity: (i) the spin frequency of the rotating and cutting wheels, which determines the speed at which the droplets are produced, and (ii) the initial diameter of the droplets [59,60]. The mechanical cutting method with a specific device is shown in Figure 3. A schematic presentation of mentioned methods is shown in Figure 4. 

## 5. Gelling Agents

Ionotropic gelation is a method based on a reaction between a polymer solution and gelling agent. As a result, it is possible to compound semi-solid gel which has optimal physicochemical and pharmacological parameters [61,62]. A significant role in ionotropic gelation plays choosing the right ionotropic medium. Both the various monovalent, divalent, or trivalent cations, as well as the concentration of the chosen agent, have an impact on the gelation rate [63], degree of derivatization [61], drug encapsulation efficiency [64], drug loading, beads size [65], circularity [66], mucoadhesive properties [25], ability to swell in aqueous environments [20]. As Bera et al. showed the gelation rapidity depends on the entropic benefit of accommodating a single multivalent ion, which offers equal electrostatic shielding as obtained by multiple monovalent ions. This is due to the encompassing of an extra positive charge, which can be found in trivalent ions as compared to divalent [63].

The choice of the ion affects not only the gelation rate, as shown by Kulkarni et al., who also confirmed the influence on the beads stability. Crosslinking of gellan gum is more effective for multivalent cations—calcium, magnesium, and zinc ions give more stable beads than monovalent sodium and potassium ions [62]. In addition, with the use of calcium ions, compared to monovalent potassium ions, while maintaining the same ionic strength of the gelling solution, it was possible to obtain a higher percentage of spherical particles [67].

In their research, Agnello et al. proved that monovalent ions (such as Na^+^) can form formulas weaker than those obtained using divalent ions (such as Ca^2+^) [61]. The use of different divalent cations such as Mg^2+^, Ba^2+^, Ca^2+^, Cu^2+^, Zn^2+^ (1.0 M) and additional concentration of Ca^2+^ ions (2.5 M; 5.0 M) has been tested for drug encapsulation efficiency by Singh and Kin [64]. As the researchers proved in their work, the selection of an appropriate gelling agent should depend on the physicochemical properties of the API used in a formula. All of the used divalent ions had a significant effect on the aqueous solubility of API (in comparison with deionized water). What is more, the concentration also had an effect on the same parameter. Moreover, the researchers confirmed that the different ions influence the drug loading efficiency of gellan gum. The highest value of encapsulation efficiency has been obtained with the use of Cu^2+^, then with the following order: Zn^2+^, Ca^2+^, Mg^2+^, Ba^2+^. It was also observed that the drug loading parameter increased with the atomic number of the cation (except for barium). This may suggest that electropositivity plays an important role in ionotropic gelation. Researchers suggest that higher drug loading obtained with copper ions in comparison with calcium ions may arise from their effects on the sol–gel transition of the gellan gum [64]. What has also been noticed is that although transition divalent cations (Zn^2+^) exhibit higher binding energies to carboxylate anions than the alkaline earth metal cations (Ca^2+^), which can facilitate the formation of stronger coordination—covalent bonds with biopolymer chains, gelation and aggregation occurred primarily via ionic bond formation between divalent Ca^2+^ ions and biopolymer chains [63]. Although the above-mentioned advantageous properties resulted from the use of calcium or copper ions, it was magnesium ions that were used to prepare gellan gum beads with yeast cells (*Saccharomyces* sp.) encapsulated in them. Yeast cells have retained their biotechnological properties—high fermentation efficiency. Thanks to magnesium ions, it was possible to compound chemical and morphological beads with *Saccharomyces* sp. with high metabolic activity. Another advantage of this technology is the multiple uses of compounded beads [68].

As already mentioned, not only divalent ions can be used as gelling agents. As the divalent ions can conjugate only four anionic groups of two different gellan chains, the trivalent ions, because of their higher electropositivity potential, can conjugate more carboxylate groups belonging to glucuronic acid molecules in the gellan chains. Due to this property, trivalent ions can be advantageous by enabling a faster crosslinking reaction (when compared to divalent ions). Moreover, it is possible to use a lower concentration of crosslinking solution to obtain the formation of more rigid beads, so we can reduce the risk of drug solubilization or degradation in the crosslinking solution by increasing encapsulation efficiency [66,69,70].

In another work, it has been proven that the use of different concentrations did not have a significant impact on the particle diameter. In this work, the effect of different calcium chloride concentrations on swelling in aqueous environments was shown. In general, the swelling degree reduces as the concentration of cross-linker rises [20]. As it turned out, both the particle size of beads and their circularity depended on polymer content and crosslinker concentration. Increasing the concentration of AlCl_3_ from 3% to 5% promoted a rise in particle size (a larger number of Al^3+^ ions can build a more branched network, which relates to enlarging particle size). The circularity also depended on the polymer content and crosslinker concentration. Various values of the above variables were investigated in terms of creating spherical-shaped beads. The best circularity was shown for the 2% polymer content associated with the high crosslinker concentration—5%. On the other hand, increasing the Al^3+^ ions concentration caused more irregularity on the surface of the bead [69].

The method of ionotropic gelation of gellan gum can be applied to prepare a dosage form with high mucoadhesive properties. Prezotti et al. [70] prepared particles (with aluminum chloride used as a gelling medium) characterized by high mucoadhesivity, which contributed to the improvement of release parameters. In this case, ketoprofen release was also pH-dependent and controlled. It was also shown that the increase in the concentration of the gelling solution favored an increase in the particle size. The controlled release has a high significance in the eradication of bacterial diseases. By using gellan gum and calcium ions beads with encapsulated amoxicillin with an optimal shape and size were received. The particles were additionally coated with chitosan to avoid erosion in the stomach. In this case, the obtained product revealed high adhesiveness to the gastric mucosa, and its eradication effectiveness against *Helicobacter pylori* was assessed in vitro. The investigated dosage form was found to be more effective than the conventional products revealing non-modified drug release [25].

## 6. Solid Oral Dosage Forms

More complex delivery systems are distributed more uniformly in the gastrointestinal tract, and in this way, the irritation can be reduced. Babu et al. [65] investigated the possible use of gellan macro beads as a carrier for a poorly soluble active ingredient, amoxicillin. In the investigation, a controlled release system with gellan gum and amoxicillin was prepared by ionotropic gelation with calcium ions. They investigated the effect of polymer or electrolyte (CaCl_2_) concentration, drug loading, curing time, stirring time, etc. influencing the preparation of the gellan gum macro beads and the drug release from prepared beads. The success of their investigation was the preparation of beads with high incorporation efficiency for amoxicillin (91%). As was shown, when the percentage of the polymer (from 1% to 3%) and drug (0.5% to 2%) increased, the diameter of beads also increased. This could be explained by surface tension influencing the size and shape of beads. Surface tension causes the formation of spherical droplets, while gravity elongates them vertically. Decreasing the surface tensions leads to a decrease in the size of forming droplets [71]. This effect could be also explained by a slower flow of the more viscous gellan solution out of the syringe. As a result, the drops and then the beads had a larger diameter. Moreover, soft and sticky beads were observed when a theoretical loading of amoxicillin was at the amount of 2%. In this case, it was also possible that amoxicillin, could partially complex the negatively charged functional groups of gellan gum, and in this way, there was no place for the Ca^2+^ ions. The investigation showed that the gellan beads stay stable at a low acidic pH, but they swell and quite easily disintegrate at a higher pH [15]. Both swelling and disintegration of prepared beads were dependent on the pH of the dissolution medium as well as the solubility of the drug entrapped in the gellan beads. The burst effect of amoxicillin from beads was observed in all three media. This effect may occur because of the presence of the crystals of the drug on the surface of the bead or in the case of unequal distribution of the active ingredient inside the gellan beads. It is likely that the initial high rate of the release was observed since the water absorption by the gellan beads during the dissolution of amoxicillin is radial inwards. The rate decreased during the time period because the diffusional path length was longer and probably because of the lower content of amoxicillin in the bead center. The investigation of the release kinetics of amoxicillin from gellan beads presented by Babu et al. [65] showed that release followed the diffusion model for an inert porous matrix in the order: 0.1 N HCl > phosphate buffer > distilled water. According to the higher solubility of amoxicillin at a lower pH, the released in 0.1 N HCl was higher than that in phosphate buffer. The shape of the gel beads did not change in appearance, even at the end of the drug release study. The researchers suggested that the gel structure was maintained throughout the release process and that the rapid drug release from gellan beads in the low pH could also be explained by a change in the matrix properties upon contact with acid. The conclusion was that drug concentration, polymer concentration, and electrolyte concentration significantly affect the release rate of amoxicillin from the beads. The gellan macro beads could be suitable for gastro-retentive controlled delivery of amoxicillin.

Moreover, another group investigated the use of ionotropic gelation for the preparation of gellan beads containing the acid-soluble drug amoxicillin trihydrate [25]. The purpose of the study of Narkar and co-workers was the development and evaluation of a mucoadhesive drug delivery system for the controlled release of API in the stomach. The study showed that the use of the alkaline crosslinking medium in the preparation of gellan beads had higher entrapment efficiency than those with the acidic crosslinking medium. The entrapment efficiency was in the range of 32% to 46% *w*/*w* and 60% to 90% *w*/*w* in acidic and alkaline mediums, respectively. Additionally, batches with the lowest, medium, and highest content of the drug were subjected to chitosan coating to form a polyelectrolyte complex film. It was observed that the drug entrapment efficiency and particle size increased with the polymer concentration. Scanning electron microscopy showed a spherical but rather rough surface due to the leaching of the drug in an acidic crosslinking solution, a dense spherical structure in an alkaline crosslinking solution, and a rough surface of chitosan-coated beads with minor wrinkles. The in vitro drug release study showed that up to 7 h release of active substance followed in a controlled manner according to the Peppas model (r = 0.9998). In vitro and in vivo mucoadhesive studies showed that beads had good mucoadhesivity, and more than 85% of beads remained adhered to the stomach mucosa of albino rats even after 7 h. The complete eradication of *Helicobacter pylori* was observed in an in vitro growth inhibition study. The results indicate that a mucoadhesive system based on gellan gum for controlled release of amoxicillin in the stomach may be useful in *H. pylori* treatment.

Some active substances could cause irritation of the gastric mucosa, are unstable in gastric pH, or possess small bioavailability, which is the reason for investigations focusing on drug delivery to the intestine. One of these studies was proposed by Bhattacharya et al. [72], who developed the hydrogel microbeads with the delayed release of tranexamic acid (TA) to avoid repetitive dosing of the drug and dose-related toxicity related to the conventional dosage form administration. On the other hand, TA causes gastric upset upon its release in the acidic environment of the stomach. Gellan gum microbeads were prepared by a classic sol–gel transition induced by the ionic crosslinking method using aluminum chloride as a crosslinking agent. The swelling studies of the beads showed that the increase in polymer concentration in the microbeads increased water uptake. In the in vitro release studies, in an acidic medium, less than 20% of TA was released during 2 h of the experiment. Complete drug release was achieved in an alkaline medium at different periods, depending upon the process variables, and the process lasted up to 8 h. The release followed a non-Fickian type transport mechanism. The researchers concluded that the results of the study indicated that drug-loaded hydrogel microbeads could be used to minimize the release of TA in an acidic medium and modulate the drug release in an alkaline medium. Such results suggested that formulation would help to minimize the loss of the drug due to first-pass metabolism and enhance bioavailability.

Another example of the preparation of gellan gum-based hydrogels with the ionotropic gelation method was proposed by Allam and Mehanna for the controlled release rate of metformin (MT) [73]. The obtained gellan beads were spherical in shape with a less rough surface, and the manufacturing process was characterized by high yield and entrapment efficiency. In the presented study, the in vitro drug release tests were performed for 8 h. Release profiles of MT from beads were compared with the release profiles of MT from commercial tablets. Immediate-release tablets gave 100% release after 1 h, while the retarded product (gellan beads) released 100% of the active substance within about 3 h. The authors concluded that the prolonged release of metformin could be explained through a range of factors including the swelling of the biopolymer and the ionic interaction between the drug and the GG and microbeads. Considering the results of the study, it may be assumed that the investigated novel dosage form exhibited potential as an oral sustained-release MT system.

Different connections of gellan gum with some other polymers were used in further investigations proposed by diverse groups. A remarkably interesting direction was the use of gellan beads prepared by ionotropic gelation for colon-targeted delivery. Subsequently, a successful colon-specific drug delivery system should protect the encapsulated drug against the low pH and enzymes of the stomach, preventing the drug release in this organ, and should release it upon contact with a higher pH, in the colonic environment. Such investigations were prepared, e.g., by Boni [66] and Prezotti [19,70], who worked in the same research group.

In the first study [66], gellan gum microspheres preparation by the ionotropic gelation technique, with the use of trivalent ion Al^3+^ (aluminum chloride as gelling agent), was presented. Ketoprofen was used as the active substance. Further investigation was focused on the composition of gellan gum and pectin as the polymers forming beads for ketoprofen or resveratrol [19,66]. In the investigation presented by Boni and co-workers [66], the percentage of entrapment efficiency ranged from 49% to 87%. In the twenty-two randomized experiments performed in a full factorial design, it was shown that the increase in polymer concentration as well as the decrease in crosslinker concentration positively influenced the amount of encapsulated drug. The size of the microbeads and circularity index indicated the range from 700 to 940 μm and from 0.641 to 0.796, respectively. It was observed that the increase in polymer concentration (from 1% to 2%) and crosslinker concentration (from 3% to 5%) caused the enlargement of particle size and circularity, consistent with the previous investigation described by Babu and co-workers [65]. On the other hand, increased crosslinker concentration and reduced polymer content made the particles more irregular. As shown, macrobeads possessed high mucoadhesivity and high liquid uptake ability, and the pH variation did not affect this parameter. In the in vitro release test, the pH-dependent release of the drug was observed. The release rates in acidic pH were 42–45%, and in further investigation in phosphate buffer pH 7.4, the burst effect was observed. The entire amount of the drug was released for up to 4 h. The Weibull model had the best correlation with the drug release data, demonstrating that the release process was driven by a complex mechanism involving the erosion and swelling of the matrix or by non-Fickian diffusion. 

The other part of the Investigation of this group was presented by Prezotti and co-workers [70]. Their work was concerned with the use and characterization of biodegradable mucoadhesive beads prepared with gellan gum:pectin (GG:P) intended for the controlled release of ketoprofen (KET) [70] or resveratrol (RES) [19]. In both investigations, beads were prepared by an ionotropic gelation technique, by dripping the negatively charged polymer dispersion containing active substances into a crosslinking solution containing the positively charged aluminum ions. The researchers developed beads with a circular shape (circularity index of 0.730–0.849 and 0.81 for KET and RES beads, respectively) and particle size of 728–924 µm and an average of 914 µm for KET and RES beads, respectively. The in vitro drug release study showed that beads were able to reduce release rates in gastric media and control release at an intestinal pH of 7.4 for up to 8 h for ketoprofen, or at pH 6.8 for 48 h, for resveratrol. It was observed that beads were able to significantly reduce release rates in acid media compared to the free drug. Taking the in vitro release profiles recorded for API-loaded beads in acidic media (pH 1.2) into consideration, it was observed that 20–34% of KET or 18% of RES was released after 2 h from the beads, while almost 70 or 85% of free API was already dissolved at the same time for KET or RES, respectively. In the KET-beads investigation, the researchers observed that in pH 7.4, the burst effect was observed in the first 40 min. Drug release was completed after 3 h and up to 6 h, depending on the composition of Gellan: pectin. Free RES completed its dissolution in 2.5 h, right after changing the pH to 6.8. However, GG:P beads were able to control drug release for up to 48 h without any burst effect. An evaluation of the release kinetics modeling showed that the ketoprofen release data correlated better with Korsmeyer–Peppas for both media, while Weibull’s model correlated better with release data of RES. The study indicated that the release process was driven by a combination of Case II transport or anomalous for ketoprofen [70] or Fickian diffusion and Case II transport for resveratrol [19]. The researchers concluded that diffusion and swelling/polymer chain relaxation contributed equally to control drug release rates. The impact of the polymer carrier system on the cytotoxicity and permeability of RES was also evaluated [19]. In the cytotoxicity experiment, researchers observed that beads and isolated polymers were safe for Caco-2 and HT29-MTX intestinal cell lines. Additionally, the encapsulation of RES into the beads allowed for an expressive reduction in drug permeation in an in vitro intestinal model. Based on this feature, associated with low RES release rates in acidic media, it was concluded that the investigated beads could favor targeted drug delivery in the colon, which is a promising behavior to improve the local activity of resveratrol. 

The other examples of gellan beads as a drug delivery system for non-steroidal anti-inflammatory drugs (NSAID) were proposed by Osmałek et al., who investigated meloxicam (MLX)- and naproxen (NPX)-loaded gellan beads [15,74]. In both studies, calcium chloride was used as the gelling agent in ionotropic gelation. 

The first study was based on the computer-aided design and evaluation of a novel gellan-based oral dosage form for the modified release of MLX. The influence of drug, polymer, and surfactant content on the properties of the beads obtained under constant conditions was investigated. The results showed that the parameters, i.e., active substance encapsulation efficiency, swellability index as well as in vitro drug release depended on the composition of the investigated beads. The study showed that the concentration of the polymer affected the morphology of the obtained matrices, while the surfactant concentration had an impact on the drug loading efficiency. Swelling and dissolution studies indicated that the dosage system based on gellan gum revealed pH-dependent behavior, which could be potentially applied in MLX delivery to distal parts of the gastrointestinal tract, e.g., colon delivery. The release profiles were fitted to the Korsmeyer–Peppas kinetic model equation. The researchers concluded that the rate of drug release was controlled by the balance between drug diffusion driven by the concentration gradient, polymer relaxation due to water uptake, and the osmotic pressure in the polymer matrix.

In the next study, gellan microbeads evaluated by Osmałek et al. contained NPX [15]. The results presented in their research indicated that NPX crystals could be effectively encapsulated into the gellan gum with the ionotropic gelation method. The study also investigated the influence of the addition of carrageenans, guar gum, cellulose sulfate, and dextran sulfates on the gellan gum beads properties. The researchers observed in the swelling study that the investigated matrices tend to swell and erode at pH 7.4, which may also contribute to the increased drug release rate. The observations made for the acidic conditions were inconclusive. The beads seemed to be resistant to pH 1.2 but swelled significantly at pH 4.5. However, the latter value may be considered as non-significant regarding physiological conditions. The release process was the most efficient for series prepared with the combination of gellan gum and cellulose sulfate and after 2 h reached almost 20%. The slowest release of NPX was observed for the beads prepared from pure gellan or the blend with guar gum. 

For both evaluations, the results of drug release and swelling studies indicated that the prepared systems revealed pH-dependent properties which could be used for the modification of the drug release process in the GIT.

Additionally, Gadziński and co-workers [1] investigated the use of gellan gum in combination with different polymers as a potential oral dosage form with the ability to protect the drug from the acidic conditions of the stomach. Another feature of the proposed systems was the sustained release of the drug, allowing for potential shifting of the therapeutic effect to the colon. They used roxithromycin (ROX) as the active substance, and calcium chloride was used as a crosslinking agent. The study showed that the combination of gellan and methylcellulose or κ-carrageenan in matrices released ROX slower at pH 7.4 than the other matrices. The polymer matrices remained physically stable at acidic pH similar to the environment of the stomach. However, in acid conditions degradation of the active substance was observed, which indicates the inevitability to further modify the applied technology. Interesting results were observed when the beads were immersed at pH 7.4; the drug release experiments clearly showed that by mixing the gellan with other natural polymers, the release pattern could be changed, which might be promising in terms of colonic drug delivery. The researchers observed the best results in terms of the drug release modification for the matrices containing gellan/methylcellulose and gellan/κ-carrageenan mixtures. Additionally, the observed drug release profiles corresponded to the swelling behavior recorded for the investigated formulations. As it was shown, only 20–30% of the active substance was released from the beads after 1 h at pH = 7.4. It was related to the weakest swelling ability of formulation. This might explain the slower water penetration to the internal parts of the beads and also slower drug diffusion. Received results of the investigation showed that the evaluated complex polymer matrices could be used as modified-release dosage forms with the potential ability to release the drug at least partially in the colon, which could be advantageous in the treatment of the inflammatory conditions localized in the distal parts of the GI tract.

Another example of the crosslinking agent was evaluated by Jana and Sen [75] for the interpenetrating network micro-beads formulated with the use of two biopolymers such as gellan gum and PVA. They used zinc chloride and GA as a cross-linker. For the model drug, the researchers used aceclofenac (AC). The idea of preparing microbeads was aimed at prolonging the time of action of an active substance. A series of studies showed that prepared microbeads were spherical in shape and incorporated the drug within the compatible polymer matrix. The proposed gellan gum/PVA micro-beads system seemed to be a promising carrier for the delivery of drugs for a prolonged period. An in vitro release study revealed that microbeads with a polymer ratio of 1:1 released about 68% of AC in a sustained manner in 6 h at pH 6.8 (phosphate buffer). 

In the study proposed by Adrover and co-workers [20], gellan gum (GG) was used to formulate the spherical porous beads suitable as sustained drug delivery systems for oral administration of theophylline and cyanocobalamin (vitamin B12). GG was crosslinked with calcium ions to prepare polymeric beads. Additionally, beads were formed with and without the presence of the synthetic clay laponite, and the prepared formulations were freeze-dried. As was shown, the swelling degree of the beads was lower in SGF (simulated gastric fluid, HCl 0.1 M) than in SIF (simulated intestinal fluid pH 7.4), and it was further reduced in the presence of laponite. The presence of laponite in the formulation increased the drug entrapment efficiency and decreased the release kinetics of both drugs in the gastric environment. The authors proposed a moving-boundary swelling model with a “diffuse” glassy–rubbery interface in order to describe the swelling behavior of porous freeze-dried beads. From the release data, it was observed that the release model from the highly porous beads was different for the used drugs. Release curves of theophylline exhibited a typical Fickian behavior, while vitamin B12 release curves were influenced by the physical/chemical interaction of the polymer/clay and active substance complex. It was concluded that laponite may be an effective supplement for preparing sustained drug delivery systems based on gellan gum. 

An interesting investigation was presented by Cardoso et al. [76], who evaluated ionic crosslinking (IC) and dual crosslinking (DC) (ionic/covalent) processes for the evaluation of novel microparticles based on gellan gum (GG)/retrograded starch (RS) blends. The authors investigated the effect of crosslinking processes on the physicochemical and functional properties of the beads and the release of ketoprofen which was an active substance. Studies showed that the prepared beads were spherical, and the particle size ranged from 1016 to 1384 µm. Additionally, both crosslinking processes increased the thermal stability of the prepared microparticles. In order to restrict the digestion of microparticles by pancreatic α-amylase, the 50% addition of resistant retrograded starch was applied. Both types of crosslinking (IC and DC) associated with high polymer concentration limited the microbeads swelling at different pH values (1.2, 7.4, and 6.0), and it was found that the increased gellan concentration (2%) improved the mucoadhesivity. The in vitro release study showed that in acidic media 14–26% of the drug was released. Different controlled drug release rates were achieved in phosphate buffer depending on the composition of the beads. The researchers concluded that the erosion of the sample observed during the swelling test was a critical parameter for enhancing the drug release rates, and the proposed blends of polymers for the preparation of beads were the potential platform for colonic drug delivery, attending to different therapeutic needs.

Some other investigations concerned with the improvement of intestinal stability and pH-sensitive release of active substances in the GI tract were proposed by Dey and co-workers [77]. They prepared pH-sensitive hydrogel beads of gellan gum loaded with quercetin. They used the ionic crosslinking technique using calcium chloride (CaCl_2_) as crosslinking agents. The important finding of the study of Dey and co-workers was the formation of stable hydrogel beads at an exceptionally low concentration of calcium chloride: 0.3% *w*/*v*. The study showed the sustained release of quercetin in simulated intestinal fluid. The prepared beads showed a pH-responsive swelling behavior with maximum swelling at a pH of 7.4. The release of the drugs was about 7, 8, 6, and 4% in 2 h with an increase in the amount of drug from 20 to 50 mg in acidic pH conditions. In the intestinal medium, the beads released 28, 32, 29, and 27% of the drug in 32 h with an increase in the amount of the drug from 20 to 50 mg. An evaluation of the release kinetics modeling indicated non-Fickian or anomalous drug transport (Korsmeyer–Peppas equation), which means that the release of the drug was controlled by both swelling and diffusion. Quercetin belongs to unstable substances, which is why their stability in GIT was also evaluated. The chemical stability of quercetin in free form and entrapped in the hydrogel matrix was determined in both SGF and SIF. The remaining amount of quercetin present was determined in SGF and SIF during the 8 and 48 h periods, respectively. After 8 h in SGF, the amount of quercitin left in the aqueous solution and hydrogel beads was 57 and 99%, respectively. After 48 h in SIF, the amount of active substance left in the aqueous solution and hydrogel beads was 24 and 50%, respectively. The findings of the study indicate that the hydrogels could improve the chemical stability of quercetin in the gastrointestinal tract [77].

The preparation of gellan microbeads occurred interesting for using design, optimization, and evaluation experiment [66,74]. In their work, Shirsath and Goswami [78] investigate the DoE of vildagliptin (VLG)-loaded gellan gum (GG): sodium alginate (SA) beads for sustained release delivery. In the study, the VLG beads were prepared by the ionotropic-gelation method with calcium chloride as a gelling agent. Thirty-two runs within response surface methodology were used to optimize the effects of independent variables such as gellan gum to sodium alginate ratio (X1) and the concentration of calcium chloride (X2%) on dependent variables such as entrapment efficiency (EE) (Y1%) and drug release (DR) (Y2%). The study showed that the %EE was in the ranges of 76–91%, and prepared beads release the drug in a sustained manner in the range of 76–85% for the duration of 12 h. The optimized beads showed pH-dependent swelling and good mucoadhesivity. The VLG-loaded GG-SA beads were successfully prepared and seemed to be promising for sustained release action for a 12 h duration. Release data were also fitted to drug release kinetic models. The best fit was found for the zero-order dissolution model, as the plot showed a maximum linearity regression coefficient [78]. 

Cardoso and co-workers [79] used polyelectrolyte complexation for the formulation of nanoparticles (NPs) based on gellan gum (GG) and chitosan (CS) with polymyxin B (PMB) as an active substance. The influence of the mass ratio of polymer and the sequence of combining the components were assessed on the formation and physicochemical properties of the NPs. As it was shown all nanoparticles possessed high positive Zeta potential (> +30 mV), which ensured electrostatic stability. The particle was regular in shape and size depending on the order of dripping. Nanoscale structures (575–974 nm) were formed when gellan gum (0.5–3 mg) was dripped into the chitosan dispersion (0.75–4.5 mg); however, aggregation of the particles (sizes > 5000 nm) occurred when CS was dripped in the GG dispersion. The study showed the particles demonstrated mucoadhesive capability. In the in vitro release study, in the first 15 min of the test, the burst release was observed for all samples. Approximately 9–19% of PMB occurred in the dissolution media. The researchers suggested that it was attributed to the release of PMB non-entrapped or adsorbed on the NP’s surface. In general, polymyxin B release was pH-dependent, and a high concentration of gellan (2 mg/mL) provided more effective control of the drug release rate. Nanoparticles prepared with the ratio GG:CS 2 mg:4.5 mg contained 1 or 3 mg of API released the lowest amount of drug in HCl 0.1 N (pH 1.2), and the drug release rate showed a controlled manner in a phosphate buffer 0.1 M (pH 6.8). It was concluded that these NPs were found to be more suitable for colon-specific PMB delivery. The important results of this examination suggest that nanocarriers with matched properties can be used to overpass the challenges of oral administration of peptides, such as PMB, contributing to the progress in the search for alternatives to the oral administration of PMB. The examples of oral beads produced by ionotropic gelation are presented in Table 1.

## 7. Chemical Crosslinking: Mechanisms and Agents

Despite numerous studies describing the attempts to improve the drug delivery systems intended to be administered via parenteral and transdermal routes, oral formulations remain the most popular and versatile ones. Among different oral products, solid dosage forms including tablets and capsules are mentioned as preferred by patients, mostly due to the convenient administration and accurate dosing [81,82]. It is also important to note that solid dosage forms are considered cost effective, stable, and relatively easy to handle in terms of technological processes which are well-known and understood, as these products have been manufactured for decades. A wide selection of currently available excipients and technologies enables the design of formulations with precisely tailored drug release properties, delivering the active ingredient to a specific site. In this way, the most important goals of pharmacotherapy can be achieved, i.e., therapeutic efficacy with minimized risk of side effects. 

Gellan gum as an excipient in solid oral dosage forms has been investigated for its interesting properties, including swelling ability and pH and cation sensitivity, as well as non-toxicity and biodegradability [83,84]. In the analyzed formulations, the polymer is usually employed as a matrix-forming agent in order to modify the drug release in the gastrointestinal tract. Gellan gum is a strongly hydrophilic polymer swelling strongly in polar environments, which makes it useful in prolonged-release formulations. On the other hand, it was noted that gellan gels are less susceptible to swelling and physical degradation in gastric fluid compared to the intestinal one which is related to ion sensitivity [65,77,85].This phenomenon can be utilized in the enteral formulations, which are supposed to pass through the stomach in an unchanged form and release the drug in the duodenum. Another important property of gellan gum affecting its behavior in physiological conditions is sensitivity to biodegradation induced by the enzymes secreted by the bacteria present in the colon [19]. Therefore, the polymer is also an interesting excipient that can potentially enable selective drug delivery to the distal parts of the gastrointestinal tract which can be utilized particularly in some inflammatory conditions located in the colon or colorectal cancer [86,87]. It is also noteworthy that the properties of gellan gum can be easily tuned to individual needs and expectations through various techniques, including coating with another polymer, blending with other macromolecular compounds or chemical modifications, including chemical crosslinking occurring as a result of covalent bonds formation. Chemical modifications of gellan are usually applied in order to alter its mechanical stability and gelation temperature. It is also noteworthy that covalently crosslinked hydrogels reveal slower physical degradation in physiological fluids, which may affect the drug release rates [88]. Apart from simple crosslinking, leading to the formation of intra- or intermolecular bridges, gellan is also frequently grafted with other polymers changing its original properties. Depending on the type of grafted polymer, the obtained product may reveal better resistance toward gastric acid and altered properties impacting the drug release rates. 

Over the years, the chemistry of natural polymers has become a significant branch of pharmaceutical technology. Biomaterials made from proteins, polysaccharides, and biosynthetic polymers are very promising for application in medical purposes. They are usually biocompatible, widely available, and inexpensive, and they reveal the ability to degrade in the human organism into substances that are not harmful and come from natural, green sources. It is often assumed that the unlimited possibilities of obtaining synthetic polymers with properties adapted to specific requirements and pharmaceutical and biomedical applications are sufficient and can ensure the optimization and achievement of specific parameters by selecting appropriate substrates and properly conducting the synthesis process [89,90]. Unfortunately, for pharmaceutical purposes, the materials must meet a number of strict requirements among which the most important are biocompatibility, as well as lack of toxicity and carcinogenicity [91,92]. These are difficult to obtain in the case of synthetic polymers, mostly due to the presence of monomers, low-molecular fractions, or solvent traces hard to remove by purification techniques [93]. On the other hand, there is a huge area of use as biomaterials and pharmaceutical raw materials based on natural polymers. Although physical crosslinking usually leads to materials with low mechanical strength, thanks to chemical crosslinking methods, it is possible to obtain products that are adequately resistant and stable in a changing environment of the system. Several techniques and methods for stabilizing gellan gum by chemical crosslinking are described in the literature. Many of them are related to obtaining materials with new properties in relation to pharmaceutical applications, mostly controlled drug delivery, while others concern biomedical issues including tissue engineering and others. Chemical crosslinking can change such properties of polymer-based materials as structure, porosity, solubility, swelling, mechanical strength, thermal stability, absorption ability, susceptibility to degradation, rheological and textural behavior, etc. [94].

**Figure 5:**
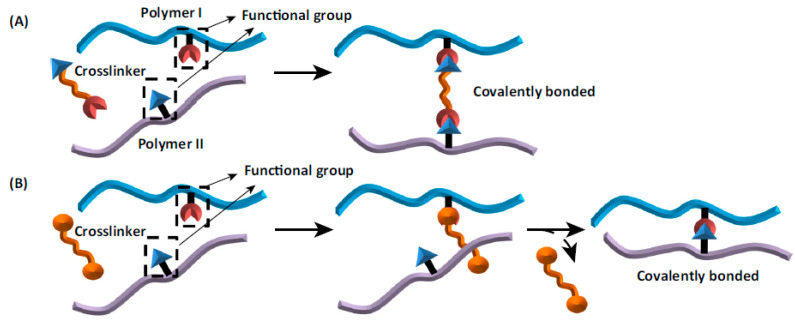
Chemical crosslinking may occur by a reaction between functional groups of polymer(s) and a crosslinking agent which acts as a covalent link between the chains (**A**) or the reaction between the polymer chains and a crosslinker which acts to connect the chains but is disposed of after the reaction (**B**) Reprinted with permission from [24]. Copyright 2022, Elsevier.

Chemical crosslinking of gellan can occur between different chains or within the same chain. In addition, depending on the type, crosslinking agents can themselves create cross-links, as well as activate carboxyl groups leading to their direct connection through ester bridges. The main challenge in chemical crosslinking not only of gellan gum but also other natural polysaccharides is the selection of appropriate reaction conditions ensuring a homogeneous product with regular distribution of the polymer network throughout the entire volume, without clusters of higher and lower density and network defects [95]. Most of the studies describe crosslinking of gellan in the entirely dissolved state. For better stabilization and homogeneity, it is also suggested to use the polymer in an initially self-aggregated state after the formation of the double helices. Satisfactory results are also obtained by the initial chemical modification of gellan chains. Factors causing the chemical gelation of gellan include sodium trimetaphosphate (STMP), GA, anhydrous alcohol environment, or radiation of the appropriate wavelength. Often, the crosslinking itself is preceded by a suitable initial modification of the gellan chains, for example, with the use of methacrylic moieties. The schematic process of chemical crosslinking is shown in Figure 5.

The chemically crosslinked polymers have already found application in various medical treatments. In situ chemically cross-linkable gellan hydrogel based on gellan thiolation designed by Du et al. [96], electrospun nanofibers made of gellan gum crosslinked with poly(vinyl alcohol) for tissue engineering [97], betamethasone-containing gellan gum hydrogels enzymatically crosslinked by tyramine for intraarticular injections in rheumatoid arthritis treatment [98], oral anti-cancer patches [99], and cross-linked hydrogels comprising gellan gum and chitosan applied in wound dressing [100] are perfect examples of the innovative medical solutions using natural polymers. However, oral drug delivery systems based on chemically crosslinked polymers are still an area for detailed research and an opportunity to develop novel tools for effective therapy. Gellan gum is one of the most promising polymers for this purpose.

Various chemical crosslinking factors have been used for the process of crosslinking, and they include the above-mentioned GA, STMP, or EDC (ethyl-3-(3-dimethylaminopropyl)carbodiimide), NHS (1-ethyl-3-[3-dimethylaminopropyl]carbodiimide hydrochloride and N-hydroxysuccinimide), epichlorohydrin, citric acid, glyoxal, and many others [24]. This chapter is going to describe the oral drug delivery systems using cross-linked gellan gum.

Among the chemical agents used for gellan crosslinking, STMP and GA are often mentioned. The first one is broadly used in the food industry as a crosslinker for several types of natural polymers. Its most advantageous property is very low toxicity. The crosslinking mechanism by STMP is the result of intermolecular phosphate link formation between polymer chains. Depending on the structure of the polymer, STMP can react with two alcoholate groups located on two separate polymer chains or on the same chain [101].

**Figure 6:**
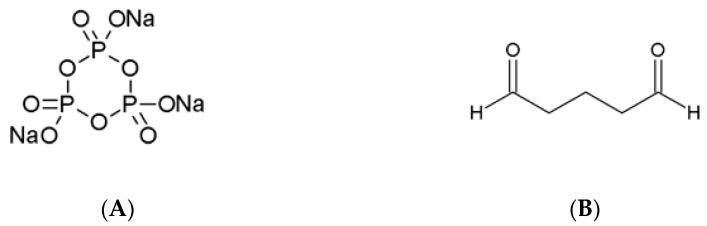
Chemical structures of STMP (**A**) and GA (**B**).

Glutaraldehyde is a highly effective chemical cross-linker with a very wide spectrum of applications. Its aldehyde groups are highly reactive and create covalent bonds with hydroxyl groups, amines, thiols, imidazoles, and phenols [101]. In an alkaline aqueous environment, GA undergoes the aldol condensation reaction resulting in the formation of -CH=C(CHO)- polymeric chains. The main purpose of using GA as a crosslinking agent is to obtain materials with increased resistance and stability to the aqueous environment while maintaining biocompatibility and the ability to release active substances in a specific place of the organism. The chemical structures of both STMP and GA are presented in Figure 6.

Joglekar et al. performed comparative studies to depict the differences between the effects related to the application of these two cross-linkers concerning degradation profile, swelling capacity, porosity, mechanical strength, morphology, and in vitro biocompatibility. The aim was to show which one can be potentially used for the fabrication of tissue scaffolds. The scaffolds were fabricated using hydroxyapatite (Hap) and polymers including gellan gum, xanthan gum, and PVA, crosslinked using either GA or STMP. Reference scaffolds without the addition of crosslinking agents were also fabricated. It turned out that crosslinking with GA resulted in lower degradation rates. According to the mechanical strength resistance toward the compressive forces, STMP-modified materials revealed better properties than GA. Additionally, the cell viability experiments showed that STMP can be regarded as a safer crosslinking agent [41]. 

Interpenetrating (IPN) microcapsules based on gellan gum and egg albumin were prepared by Kulkarni et al. for the modified release of diltiazem HCl. The drug was additionally complexed with cation exchange resin, Indion 254^®^. As a chemical cross-linker for GG and albumin, GA was used. Additionally, gellan was stabilized by Ca^2+^ ions. It turned out that in the acidic environment, the pure drug dissolved entirely within 1 h, while the complexation with resin extended the release to 9 h. The most prolonged release was observed for dually cross-linked capsules and lasted for 15 h. For all obtained microcapsules, physicochemical analyses including Fourier-transform infrared spectroscopy (FTIR), thermogravimetric (TGA), differential scanning calorimetry (DSC), and X-ray diffraction (XRD) studies were performed. Moreover, they were tested for swelling ability and in vitro drug release in simulated gastric and intestinal fluids. The obtained microscopic images indicate a smaller diameter of beads produced with the use of both crosslinking agents compared to those produced with calcium chloride alone. The observed effect can be explained by higher gel rigidity resulting from the covalent bridges in the hydrogel matrix. The concentration of calcium chloride applied in the gelation process also affected drug entrapment efficiency. At higher concentrations, drug entrapment efficiency decreased, which was most probably related to the replacement of diltiazem molecules bound to the resin by calcium cations. In the case of microcapsules prepared with both crosslinkers, the higher concentration of glutaraldehyde led to higher entrapment efficiency, which was most probably caused by higher matrix rigidity. Higher polymer rigidity due to higher glutaraldehyde concentrations was also associated with lower swelling ability and sustained drug release compared to ionically crosslinked microcapsules [102]. 

Another study describing dual crosslinking applied to obtain simvastatin-loaded polyspheres composed of gellan gum and carrageenan was presented by Kulkarni et al. [62]. Polyspheres are multiparticulate systems containing combined smaller matrices. The investigated formulation was prepared with the use of both ionotropic gelation performed with Zn^2+^ ions and chemical crosslinking performed with glutaraldehyde. The obtained systems were subjected to scanning electron microscopy analysis, drug entrapment efficiency tests, Fourier-transform infrared spectroscopy (FTIR), thermal and X-ray studies, swelling, in vitro drug release tests, and in vivo studies involving Wistar rats. The results of physicochemical studies indicate that the manufacturing process was robust and allowed for high drug incorporation efficiency. Moreover, it was found that the drug was stable after the encapsulation in the matrices, and the results of thermal and X-ray analyses revealed that it was dispersed in an amorphous state in the carrier. Similarly to the previously mentioned studies, dual crosslinking enhanced the rigidity of the gel structure, which resulted in a decreased drug release rate compared to the matrices obtained with ionotropic gelation only. The in vivo tests showed a therapeutic effect comparable to pure simvastatin after the administration of polyspheres. On the other hand, the investigated formulation had better properties in terms of maintaining the constant drug level in the plasma. 

Vashisth & Pruthi employed chemical crosslinking of gellan gum and PVA to stabilize nanofibers for tissue engineering and wound healing obtained by electrospinning technique [97]. It was indicated that without stabilization of the polymer network, the obtained materials have weak mechanical properties and poor physicochemical stability in aqueous media. Crosslinking techniques were achieved with the use of physical, chemical, ionic, and vapor techniques. The chemical one was performed with methanol and vapor with the use of GA. In the second case, the obtained gellan/PVA nanofibers were placed in a vapor chamber containing liquid GA for 24 h. The authors indicated that although crosslinking with GA influenced the increase in the mechanical strength of the fibers, it has to be taken into account that the residues of GA may increase the risk of cytotoxicity and therefore should be thoroughly removed after the crosslinking process. 

Maiti et al. designed a carrier for the controlled oral delivery of glipizide, a hypoglycemic agent used in the treatment of type II diabetes mellitus. Gellan gum beads loaded with glipizide obtained in the process of ionotropic gelation with aluminum chloride were then treated with GA solution. The formation of crosslinking covalent bonds was confirmed by FTIR spectra. Produced GA beads presented a slower release of the active pharmaceutical ingredient compared to the beads which did not undergo the crosslinking reaction. The alteration of the pH of the dissolution medium did not affect the drug release, which makes the obtained beads a promising tool for decreasing the dosing frequency and dose-related side effects [27]. Other glutaraldehyde crosslinked formulations containing ketoprofen and based on gellan gum and retrograded starch aimed to provide a colon-specific drug delivery to decrease the gastrotoxicity of ketoprofen and ensure its action in the colon [80]. 

Leone et al. prepared gellan gum coated with PVA with further crosslinking with STMP. The project aimed to obtain material for the viscosupplementation of synovial fluid in joints. The idea of using PVA was the protection of GG against degradation during sterilization, improvement of rheological properties, and maintenance of injectability. The authors used EDC to stabilize the cross-linked GG and avoid potential calcification of tissues due to its affinity to calcium ions. The obtained material showed mechanical properties similar to the marketed product Synvisc^®^ (Genzyme Europe, Amsterdam, The Netherlands). Additionally, no cytotoxic effects were observed on mouse fibroblasts [103].

Another popular crosslinking agent is epichlorohydrin (C_3_H_5_ClO). It is a highly reactive organochlorine compound and an epoxide. It was used to design an oral delivery system of sulpiride, an antipsychotic and antidepressant drug. Hoossain et al. created a semi-penetrating polymer network to form a xerogel matrix system by crosslinking gellan gum with polyethylene (oxide) (PEO) and epichlorohydrin (EPI) [104]. The crosslinking of polysaccharides with EPI involves the formation of covalent links between the carbon atoms present within the polymer chains. This reaction causes a change in GG properties, for example, mechanical strength, swelling rate, and drug release profile. Additionally, in this case, epichlorohydrin also reacts with ethylene oxide resulting in the creation of a copolymer. These solutions led to an oral DDS which enhanced the bioavailability of sulpiride, ensured its sustained release, and allowed a less frequent dosing rate.

Carbodiimide chemistry was applied to develop a gellan hydrogel with demanded properties. EDC (1-ethyl-3-[3-dimethylaminopropyl]carbodiimide hydrochloride) and NHS (N-hydroxysuccinimide) are known for their ability to form an amide bond between amino and carboxyl groups. Matricardi et al. used L-lysine ethyl ester moieties to crosslink the branches of gellan gum and simultaneously prevent the carboxyl groups of the mentioned amino acid from an intermolecular reaction between its molecules [88]. The crosslinked polymer was proven to be a good base for the creation of a modified drug release formulation because in vitro studies showed that it slowed down the release of vitamin B12. EDC was also used in order to obtain oral patches made of crosslinked gellan gum, glucosamine, and clioquinol [99] designed for the treatment of early-stage oral cancer or wound care after surgery in the later stages of the disease.

There are many agents suitable for chemical crosslinking. Some of them, mentioned above, have already found application in gellan-based oral drug delivery systems, and some show great potential or have even been used in other formulations; for example, sodium trimetaphosphate (STMP) was used to obtain a modified gellan scaffold for bone tissue engineering [41]. However, research reports regarding its application in gellan-based drug delivery systems are scarce. 

## 8. Methacrylated Gellan Crosslinking

Chemical gellan crosslinking can be performed via UV light-induced reaction. In the first step of this process, the original polymer must be chemically modified, for example, by esterification with methacrylic or acrylic acid. In the next step, the polymer is subjected to UV irradiation and methacrylic moieties form covalent bridges joining adjacent polymer chains. The materials obtained with the described procedure have been utilized in biomedical applications, including tissue engineering and regenerative medicine [105]. However, to the best of our knowledge, no oral solid dosage forms obtained with this technique have been investigated.

Matricardi et al. prepared and compared the properties of low acyl gellan gum cross-linked physically and chemically aiming at the preparation of materials for modified release of high-molecular-weight drugs, e.g., proteins, after oral administration. As the cross-linker, the authors used ethyl ester of L-lysine. It was stated that chemical crosslinking resulted in higher water uptake and lower elasticity in comparison to the physically stabilized gels. Such behavior was attributed to the fact that physical crosslinking was proceeding simultaneously with the formation of the double helices, and thus, the polymer chains were more tightly packed. In the case of chemical bonding, the process was conducted when the chains were arranged randomly. The in vitro release test showed that fluorescein isothiocyanate-dextran, used as a model for the HMW compound was released within 8 h, while from chemical gels the same amount was liberated after about a 20-fold longer time [88].

**Figure 7:**
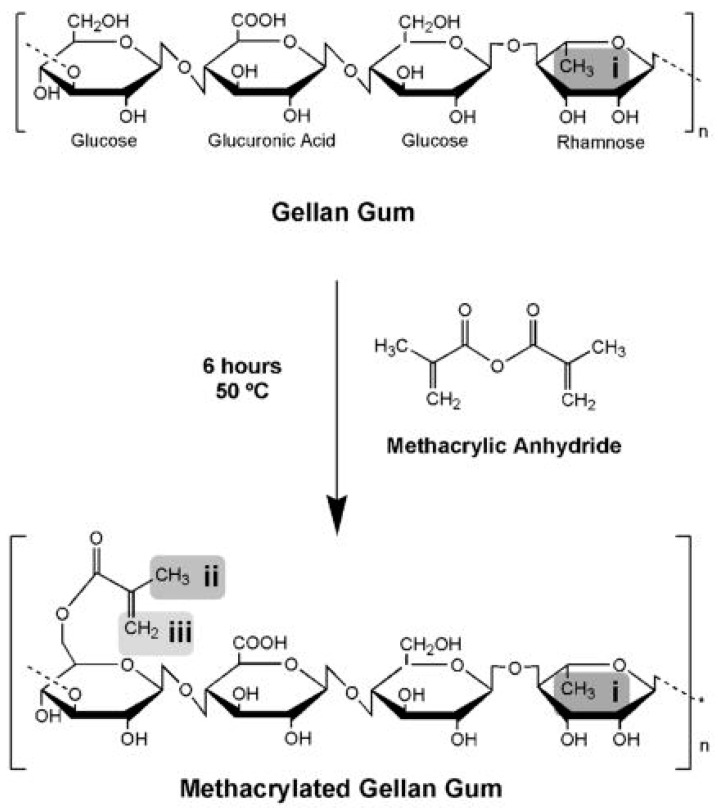
Synthesis of MeGG (i—methyl group of rhamnose, ii—methyl group of methacrylic anhydride, iii—vinyl group of methacrylic anhydride) Reprinted with permission from [106]. Copyright 2022, Elsevier.

Novel gellan-based materials for tissue engineering were prepared by Coutinho et al. The authors functionalized GG chains with methacrylate groups obtaining photocrosslinkable polymers with low and high degrees of methacrylation [106]. Calcium ions and 2-hydroxy-1-[4-(2-hydroxyethoxy)phenyl]-2-methyl-1-propanone were applied as cross-linkers. The final chemical cross-linking was obtained by exposing it to light (wavelength 320–500 nm) for 60 s. The obtained dually cross-linked hydrogels showed great potential toward the development of scaffolds for tissue regeneration. The authors showed that without affecting the biocompatibility, the mechanical properties could be adjusted to the desired requirements. Interesting observations were presented by Li & Bratlie with regard to the influence of different cross-linking mechanisms used for methacrylated GG (MeGG) toward altering macrophage response as shown in Figure 7 [107]. 

**Figure 8:**
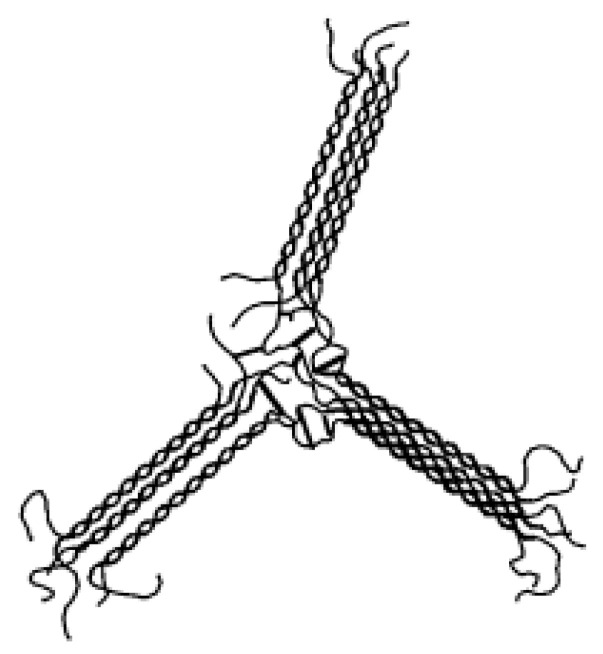
Schematic view of self-cross-linked gellan network structure, where heavy solid lines represent crosslinking points. Reprinted with permission from [95]. Copyright 2022, American Chemical Society.

A different approach has been proposed by Dentini et al. [95] The authors used partially self-ordered gellan and applied EDC for the activation of carboxylate groups on the polymer chains to form ester bonds between each other. The performed small-angle X-ray scattering analysis showed that the crosslinking points occurred between the chains which were not involved in the formation of the helical domains (Figure 8). 

Grafting, known also as graft copolymerization, is a widely applied method to modify the original properties of polymers, including the natural gums. The grafting process is defined as covalent monomers linking to a polymer backbone. In this way, the characteristics of a macromolecular compound can be improved in the desired direction, to obtain the most favorable product features. For example, Sarkar et al. [108] obtained and characterized tablets with metformin using poly (acrylic acid)-grafted gellan with the purpose of enhancing the mucoadhesive properties of natural gellan gum. The described dosage form was designed to extend the residence time in the stomach and, in this way, increase the drug bioavailability. The grafting process was promoted by microwave irradiation and was performed with the use of ceric (IV) ammonium nitrate as a redox initiator. The synthesized polymer was characterized for biodegradation and acute oral toxicity, while metformin-loaded tablets were evaluated for in vitro drug release, swelling, and ex vivo mucoadhesion. It was shown that the obtained product was nontoxic and biodegradable, even though the monomer applied in graft copolymerization is known for its toxic properties. The investigated tablets had good bioadhesive properties and released the drug in a prolonged manner compared to the reference product with non-modified gellan gum as a matrix-forming agent. A similar procedure was described by Vijan et al. [109], who obtained acrylamide-grafted gellan gum as a carrier for metformin-loaded tablets. The polymer modification procedure was the same as in the study presented by Sarkar et al. [108]. It was revealed that the introduction of additional side chains to the polymer molecule led to an increase in viscosity, and the extent of this phenomenon was correlated with grafting efficiency. The synthesized compound was generally nontoxic and biodegradable, which is promising in terms of future application in pharmacy and related areas. In vitro drug release studies indicate that the drug release rate depended on the grafting efficiency. The tablets obtained with the polymers containing higher amounts of side chains in the molecule released the drug in a prolonged manner. The same synthetic approach has been applied to attach other acrylic and methacrylic acid-derived side chains to the gellan backbone. The literature reports show examples of introducing methyl methacrylate [110], methacrylamide [111], and 2-(dimethylamino)ethyl methacrylate [112] moieties to the gellan structure. In the case of the studies aiming at the design of novel oral drug delivery systems, the obtained products were characterized by higher viscosity and extended drug release compared to the original polymer. It is noteworthy that these parameters can be modulated and tailored according to the actual needs of the number of new side chains introduced to the molecule. 

## 9. The Liquid In Situ Gelling Forms

In situ gelling fluids after oral administration provides an interesting alternative to traditional formulations. Their undoubted advantage is that they have a liquid consistency before being swallowed, and as a result of contact with the acidic environment of the stomach, they solidify into a stiff gel. As a result, they do not pass quickly in the gastrointestinal tract, releasing the drug in a prolonged manner. This solution is particularly interesting in relation to drugs that are expected to reveal therapeutic activity or be absorbed in the stomach or upper part of the small intestine. The idea is based on the preparation of gellan solutions together with the complexed gelling ions. After reaching the stomach, the ions are displaced from the complex and cross-link with the liquid polymer solution. 

**Figure 9:**
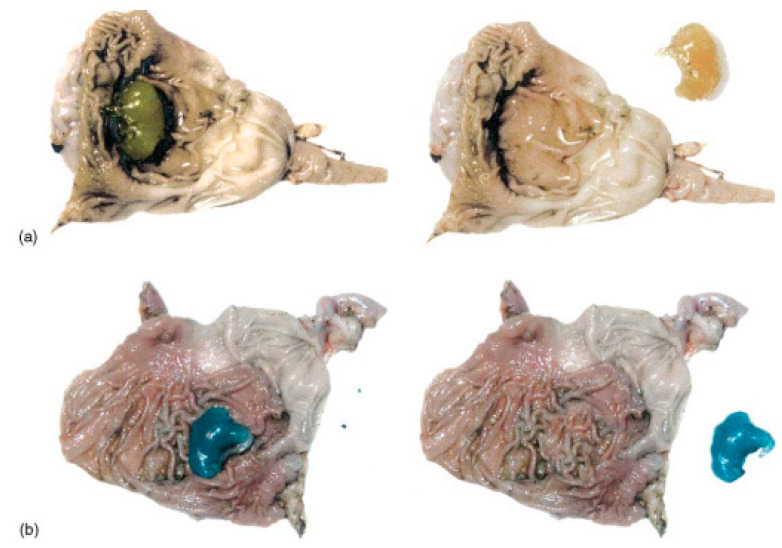
Photographs showing the presence of gels in rabbit stomach 5 h after oral administration of (**a**) a 1.0% (*w*/*v*) gellan sol and (**b**) a 1.5% (*w*/*v*) alginate sol. Reprinted with permission from [114]. Copyright 2022, Elsevier.

One of the first works related to this issue was presented by Miyazaki et al. [113]. The aim was to prepare liquid formulations with theophylline based on a 1% GG solution. Calcium chloride was used as the physical crosslinking agent, and sodium citrate was applied for its complexation. In the acidic environment of the stomach, calcium ions were released, which resulted in crosslinking of gellan. The formulations were tested in vivo and revealed a bioavailability from three- to five-fold higher than the commercial oral sustained release suspension [113]. A continuation of the research was the work presented by Kubo et al. The authors compared two types of in situ gelling oral formulations containing paracetamol. Sodium alginate (1.5%) and gellan gum (1.0%) solutions were used as the vehicles. Visual observation of rat stomach content (Figure 9) confirmed that alginate-based formulations remained at 52% after 5 h whereas gellan formulations showed only 18% of mass loss. It was also concluded that in situ gelling has a longer release than aqueous paracetamol solution, at a level similar to a commercial suspension [114].

The composition of gellan-based in situ gelling systems can be also modified in order to achieve the floating properties. Rao & Shelar used calcium carbonate both as a gelling agent and a source of gas bubbles. The formulations contained itopride hydrochloride, a prokinetic drug, which is used for better emptying of the stomach and supporting the food content passage. Additionally, HPMC in various concentrations was used as a release-suspending agent. The in vivo experiments on male Wistar rats proved that due to gelation in the stomach, the maximum blood concentration of the drug was reached after 6 h, while in the case of pure drug it was 1 h [115]. 

**Figure 10:**
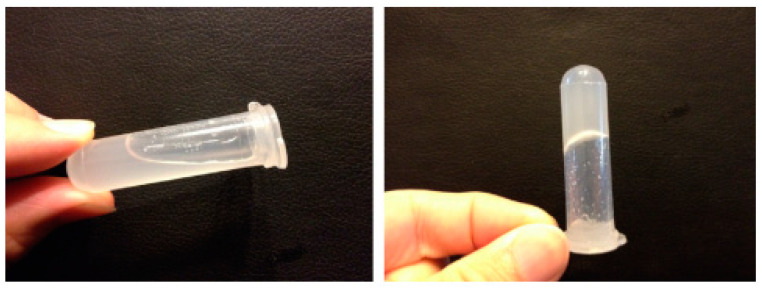
In vitro hydrogel formation with in situ gels (0.6%) and artificial tears [117].

The in situ gelling properties of gellan are not only used for oral delivery of actives to reach systemic action but also for local delivery. Since the secretions and body fluids contain various types and amounts of cations, it is possible to design gellan formulations with the ability to undergo an ion-activated sol–gel transition in the oral and nasal cavity or the surface of the eye. Harish et al. prepared a mucoadhesive in situ gelling liquid formulation containing clotrimazole for prolonged release in the oral cavity. The buccal residence time was extended both by the thickening of gellan gum and the improvement of its mucoadhesive properties by the addition of Carbopol^®^934P and HPMC [116]. Other studies presented by Zhu et al. concerned the preparation of ophthalmic formulations containing ketotifen. The concentrations of 0.25 and 0.6% of gellan showed a considerable increase in viscosity after mixing with artificial tear fluid (Figure 10) [117].

**Figure 11:**
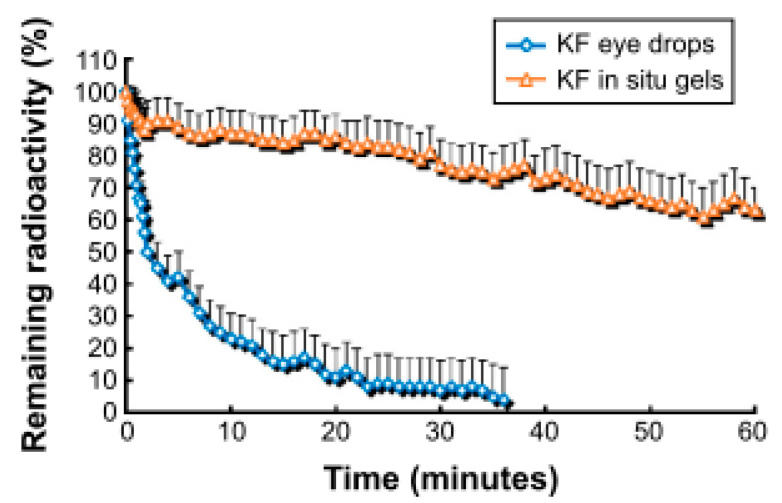
Corneal surface clearance of two formulations incorporating 99 mTc-DTPa [117].

With the use of the gamma scintigraphy technique, the authors confirmed that the clearance time of in situ gelling formulations was significantly longer than traditional viscous teardrops (Figure 10 and Figure 11).

**Figure 12:**
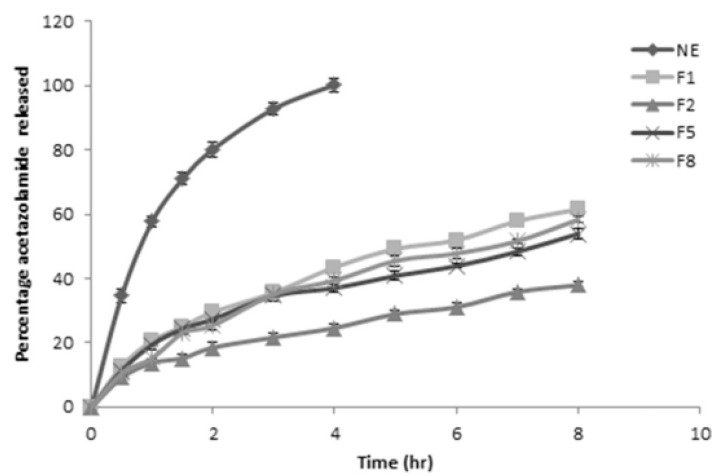
Percent of drug released from nanoemulsion (NE), nanoemulsion in situ gel gellan (F1), nanoemulsion in situ gel gellan/xanthan (F2), nanoemulsion in situ gel gellan/HPMC (F5), and nanoemulsion in situ gel gellan/carbopol (F8) Reprinted with permission from [118]. Copyright 2022, Elsevier.

Nanoemulsion (NE) in situ gelling system was obtained by Morsi et al. for the ophthalmic delivery of an antiglaucoma drug, acetazolamide. The polymer base for NE consisted of gellan alone or its mixtures with xanthan, HPMC, or Carbopol^®^. From Figure 12, it can be observed that the most prolonged release was achieved in the case of gellan/xanthan mixtures. It also turned out that the addition of Carbopol^®^ resulted in partial precipitation of the drug [118].

A different approach was proposed by Agibayeva et al. The idea was to prepare the ophthalmic mucoadhesive vehicle for the delivery of pilocarpine. The bioadhesive properties of gellan were achieved by its methacrylation and resulted in a significant prolongation of residence time. Surprisingly, the lowest degree of methacrylation (6%) provided the best adhesive properties in comparison to 14 and 49% [119]. 

Mometasone furoate in situ gelling formulations were prepared by Cao et al. aiming to deliver the drug to the nasal cavity to treat the symptoms of rhinitis. It turned out that formulations containing mixtures of gellan and xanthan gum were less prone to mucociliary clearance mechanism and remained longer in the nasal cavity than typical suspension formulations, which are not mucoadhesive [120]. Other studies for nasal delivery of salbutamol sulfate as an alternative to oral delivery of the drug were presented by Salunke & Patil. Apart from gellan, HPMC was used as an ingredient increasing mucoadhesion and modifying rheological properties. The tested formulations showed no signs of a negative effect on the sheep nasal mucosa ex vivo and released the drug in a prolonged manner [121].

After intranasal administration, it is possible to obtain a local effect, introduce the drug into the systemic circulation, as well as use the route of direct transport to the brain. The nose-to-brain pathway has recently gained increasing interest and is the subject of numerous studies, many of which concern in situ gellan formulations based on gellan [83,122,123,124]. Among many examples, the most important ones can be indicated, including the nano-gel for nasal delivery of harmine, which was developed by Huang et al. The incorporation of the drug nanocrystals in the polymer vehicle provided brain bioavailability 25-fold higher than after oral administration, which was the synergistic effect of nanonization and extension of the residence time due to solidification of the vehicle in the nose and its mucoadhesive properties [125]. Lipid-based Carbopol-gellan gum in situ nasal gel for brain delivery of teriflunomide was developed by Gadhave et al. for the treatment of glioma. Encapsulation of the drug into nano-lipid carriers provided a lack of local toxicity and irritation to the mucosa. The in vivo bio-distribution studies showed rapid drug delivery to the targeted site, with reduced liver and kidney toxicity. In vitro experiments of human glioma cells confirmed the therapeutic potential of the developed delivery system [126]. The same authors performed detailed studies on gellan-poloxamer nanoemulgels for an antipsychotic drug, amisulpride. In this case, the developed platform included the features of ion- and thermosensitivity. The animal studies on Wistar rats showed that in comparison to intranasal and intravenous administration of the drug in the form of nanoemulsion, the nano-gel was 1.48-fold and 3.39-fold more efficient in brain delivery of the drug, respectively [127]. 

**Figure 13:**
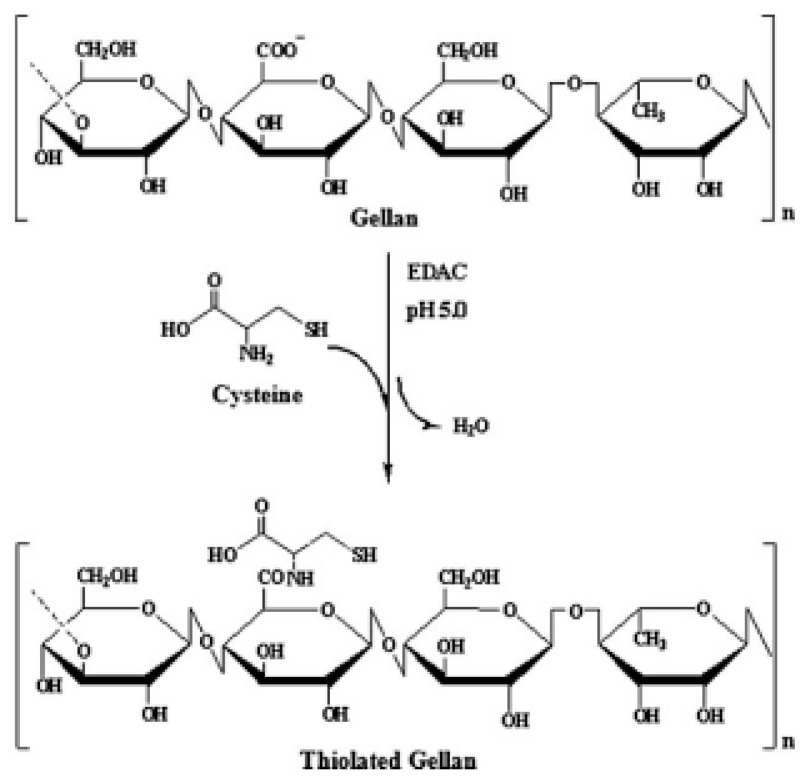
Thiolation reaction of deacetylated gellan. Reprinted with permission from [96]. Copyright 2022, John Wiley & Sons, Inc.

In addition to oral and body cavity administration, gellan-based in situ gelling systems are also being investigated for bioengineering or tissue engineering applications. Considering the not entirely appropriate mechanical properties of unmodified gellan, one of the proposed solutions is its chemical modification. In order to improve physicochemical properties, Du et al. proposed thiolation of the polymer, by the reaction presented in Figure 13. 

Due to the thiolation, lower phase transition was achieved with the maintained 3D conformation characteristic for gellan. It was shown that modification improved injectability, followed by a more rapid formation of a rigid structure. Additionally, no toxicity was observed which makes such material very promising in terms of applications for biomedical purposes [96].

## 10. Conclusions

Over the last few years, ionotropic gelation and chemical crosslinking of gellan gum have been widely exploited for the formation of novel dosage forms for drug delivery. Gellan gum-based hydrogels obtained via the mentioned methods are gaining significant interest among scientists, since potential applications thereof encompass ones ranging from solid oral dosage forms containing antibiotics or NSAIDs through semi-solids or in situ gelling hydrogels. It can be stated that both ionotropic gelation and chemical crosslinking are useful methods for the fabrication of the modified-release gellan gum-based dosage forms. However, their limitations have to be considered. Ionotropic gelation seems not to be fully effective in the protection of API against the acidic environment of the stomach. It requires an aqueous solution and a stage with an elevated temperature, which can be harmful to sensitive APIs. In turn, chemical crosslinking requires special care during the manufacturing process because of the usage of potentially toxic reagents. Both methods can be difficult to scale to industry. However, when properly prepared, they exhibit great potential with low toxicity. 

In this review, we have provided a comprehensive description of the theoretical and practical aspects of gellan gum-based hydrogels, the actual state of the art in ionotropic gelation, and chemical crosslinking methods. The most investigated gelling agents and gelling approaches and techniques were also described. The numerous references discussed in this paper are focused on the topic and should be useful for researchers interested in such scientific developments. Examples of drug delivery systems obtained by chemical crosslinking are presented in Table 2.

## Figures and Tables

**Table 1:** Examples of oral microbeads prepared by ionotropic gelation.

Crosslinking Agents	API	Addition of Polymers	Effect	Reference
calcium chloride	amoxicillin	-	release kinetics followed the diffusion model for an inert porous matrix in the order: 0.1 N HCl > phosphate buffer > distilled water	[65]
	amoxicillin	chitosan	in vitro drug release followed up to 7 h in a controlled manner	[25]
	metformin	-	100% release within around 3 h	[73]
	meloxicam	-	gellan gum beads reveal pH-dependent behavior	[74]
	naproxen	carrageenans, guar gum, cellulose sulfate, dextran sulfates	in the swelling study, the investigated matrices tend to swell and erode at pH 7.4 which may also contribute to the increased drug release rate	[15]
	roxithromycin	methylcellulose, κ-carrageenan	sustained release of API; 20–30% of active substance was released from beads after 1 h at pH 7.4	[74]
	quercetin	-	sustained release of quercetin; improvement of the stability of quercetin	[77]
	theophylline, vitamin B12	laponite	sustained release with the presence of laponite	[20]
	vildagliptin	sodium alginate	sustained release of vildagliptin up to 12 h;	[78]
aluminum chloride	ketoprofen	pectin	low ketoprofen release rates in acidic media 20–34%; control release for up to 8 h at an intestinal pH of 7.4	[70]
	ketoprofen	-	release rates in acid pH were 42–45%; in phosphate buffer pH 7.4 release up to 4 h	[66]
	ketoprofen	retrograded starch	release rates in acidic media 14–26%; controlled release in the pH 7.4	[80]
	resveratrol	pectin	low resveratrol release rates in acidic media; control release for up to 48 h at an intestinal pH of 6.8	[19]
	tranexamic acid	-	in vitro drug release less than 20% in acidic medium; controlled release up to 8 h at pH 7.4	[72]
zinc chloride	aceclofenac	PVA	sustained release of API; release about 68% of aceclofenac at 6 h in pH 6.8	[75]
polyelectrolyte gelation with chitosan	Polymyxin B	chitosan	pH-dependent behavior of PMX release from nanocarriers, reinforcing their potential for the targeted release; CS-GG complexation must have avoided the degradation of NPs in the upper GIT	[79]

**Table 2:** Examples of drug delivery systems obtained by chemical crosslinking.

Crosslinking Agents	API	Addition of Polymers	Effect	Reference
carbodiimide	Vitamin B12	-	the crosslinked polymer was proven to be a good base for the creation of a modified drug release formulation because in vitro studies showed that it slowed down the release of vitamin B12	[88]
Glutaraldehyde	diltiazem HCL	Cation exchange resin	the most prolonged release was observed for dually cross-linked capsules and lasted for 15 h	[102]
	simvastatin	-	dual crosslinking enhanced the rigidity of the gel structure	[62]
	-	PVA	GA may increase the risk of cytotoxicity and therefore should be thoroughly removed after the crosslinking process	[97]
	glipizide	pectin	produced GA beads presented a slower release of the active pharmaceutical ingredient	[27]
Glutaraldehyde/STMP	-	PVA, xanthan gum	crosslinking with GA resulted in lower degradation rates	[103]
epichlorohydrin	sulpiride	PEO	change in GG properties, for example, mechanical strength, swelling rate, and the drug release profile	[104]
STMP	synovial fluid	PVA	obtained material showed mechanical properties similar to the marketed product	[41]

## Data Availability

Not applicable.
